# Mechanism of *Escherichia coli* Lethality Caused by Overexpression of *flhDC*, the Flagellar Master Regulator Genes, as Revealed by Transcriptome Analysis

**DOI:** 10.3390/ijms241814058

**Published:** 2023-09-13

**Authors:** Guanglu Sun, Zihao Yu, Qianwen Li, Yuanxing Zhang, Mingxiao Wang, Yunhui Liu, Jinze Liu, Lei Liu, Xuping Yu

**Affiliations:** 1 Department of Veterinary Medicine, College of Animal Sciences, Zhejiang University, Hangzhou 310058, China; sunguanglu001@163.com (G.S.);; 2 Center for Veterinary Sciences, Zhejiang University, Hangzhou 310030, China

**Keywords:** *flhDC*, transcriptome analysis, oxidative phosphorylation, ROS

## Abstract

The *flhDC* operon of *Escherichia coli* encodes a transcription factor that initiates flagella synthesis, elevates flagella construction and enhances cell motility, which all are energetically costly and highly regulated processes. In this study, we found that overexpression of *flhDC* genes from a strong regulatable pN15E6 plasmid could inhibit the growth of *E. coli* host cells and even eventually cause death. We used transcriptome analysis to investigate the mechanism of *flhDC* overexpression lethal to host bacteria. The results showed that a total of 568 differentially expressed genes (DEGs), including 378 up-regulated genes and 190 down-regulated genes were detected when the *flhDC* genes were over-expressed. Functional enrichment analysis results showed that the DEGs are related to a series of crucial biomolecular processes, including flagella synthesis, oxidative phosphorylation and pentose phosphate pathways, etc. We then examined, using RT-qPCR, the expression of key genes of the oxidative phosphorylation pathway at different time points after induction. Results showed that their expression increased in the early stage and decreased afterward, which was suggested to be the result of feedback on the overproduction of ROS, a strong side effect product of the elevated oxidative phosphorylation process. To further verify the level of ROS output, *flhDC* over-expressed bacteria cells were stained with DCHF-DA and a fluorescence signal was detected using flow cytometry. Results showed that the level of ROS output was higher in cells with over-expressed *flhDC* than in normal controls. Besides, we found upregulation of other genes (*recN* and *zwf*) that respond to ROS damage. This leads to the conclusion that the bacterial death led by the overexpression of *flhDC* genes is caused by damage from ROS overproduction, which leaked from the oxidative phosphorylation pathway.

## 1. Introduction

Flagella are surface-attached appendages that propel bacteria cells to access favorable environments or to escape away from detrimental ones for better survival. In addition, the flagella also perform a variety of activities including chemotaxis, surface attachment, biofilm formation, host cell invasion and pathogenicity [1,2].

Flagella are extremely sophisticated macromolecular machines that require a lot of energy to generate, assemble, and motivate. The flagellar genes are majorly grouped as clusters in the genome [3]. Their expressions are strictly controlled as a hierarchy and coupled or coordinated with flagella assembly [4,5]. 

The flagellar genes are organized into three cascades. The *flhD* and *flhC* genes (*flhDC* for short) are early genes in the expression hierarchy and are the only two genes at the first level. Their product, FlhD_4_C_2_, is the master regulator and controls the expression of the entire flagellar regulons. The FlhD_4_C_2_ protein bound to the promoters of second-level genes, initiates their expression and fulfills the assembly of the hook basal body(HBB) [6], type 3 secretion system(T3SS) [7,8,9], etc. After the completion of HBB, the *flgM*, an antagonist of the sigma factor *fliA* [10], is secreted. The expression of the third hierarchical gene which is involved in flagellar motility and chemotaxis, is initiated and finally, functional flagella are constructed [11].

The strict regulation of flagellar expression and assembly guarantees that flagellar components are manufactured in nearly the same sequence in which they are required, enhancing assembly efficiency and saving energy consumption. In addition to their roles in the flagellar transcriptional network, *flhDC* and *fliA* are also involved in the regulation of non-flagellar genes, such utilization of dimethyl sulfoxide, and the delivery of electrons to nitrate or nitrite receptors to participate in anaerobic respiration [12,13], but some critical evidence for changes in the expression levels of target genes is lacking. In addition, most previous studies failed to accurately distinguish between direct and indirect *flhDC*-dependent regulation. Recently, studies reported that *flhDC* participated in the formation of adaptive resistance to antibiotics in swarming bacteria [14,15]. The appearance of adaptive resistance is associated with increased motility and cell adhesion [16]. This ability makes swarming an effective strategy for prevailing against antimicrobials.

The expression of the *flhDC* operon, on the other hand, is also accurately regulated. The environmental factors, such as ambient temperature, oxygen tension, pH level, nutrition type and availability, osmotic pressure, etc., regulate *flhDC* expression and flagella construction. There is a 770 bp noncoding sequence upstream of the *flhDC* operon, between the *flhD* gene and an oppositely directed *yecG* gene. This region encodes many regulatory cis-elements, in addition to promoters of the *yecG* gene and *flhDC* operon [17,18]. Most regulators binding to this region are now known to repress the expression of *flhDC* genes, e.g., the phosphorylated form of osmotic response regulator *OmpR*, the global regulator of *LysR* family protein *HdfR* [19], the activator of fimbrial expression *MatA* [20], the component of a complex phosphorelay system *RcsB* (involved in the positive regulation of capsular exopolysaccharide synthesis, biofilm formation, and acid resistance, both activates *MatA* and, in complex with *RcsA*) [21,22] and the weak negative-feedback regulator *fliZ* (on the second level in flagellar expression hierarchy, accumulates during post-exponential growth phase) [23], etc. Fahrner et al. screened mutant strains that stimulate *flhDC* expression and found that mutations at this region, even hundreds of base pairs upstream of the promoter of *flhD*, can improve the expression of *flhDC* operon [24].

Of course, there are some positive regulators activating *flhDC* expression too. For example, the global regulator cyclic AMP (cAMP)-cAMP receptor protein complex (cAMP-CRP) and the phosphorylated form of the quorum sensing response component *QseB* [25] are required to enhance *flhDC* expression. They are the only two transcription factors that directly bind to upstream non-coding sequences and activate *flhDC* gene expression. The nucleoid-associated proteins, H-NS and HU [26,27] are also positive regulators, maybe in an indirect manner. The type I fimbrial regulator *LrhA* can also derepress flagellar synthesis and increase biofilm formation [28].

In addition, *flhDC* expression is also regulated at the translational level. The transcriptional *flhDC* mRNA has a UTR of 198-nucleotide (nt) leader sequence [29,30]. It interacts with the regulator of carbohydrate metabolism, *CsrA*, resulting in increased translation. It also interacts with several other environmentally responsive small regulatory RNAs (sRNAs), including negative regulators *ArcZ*, *OmrA*, *OmrB*, *OxyS*, and positive regulator *McsA* [7,31,32]. Whether factors regulating *flhDC* expression or the downstream genes FlhD_4_C_2_ regulated, they are strictly organized and controlled to fulfill the purpose that the bacteria have appropriate and usually moderate motility to achieve the best fitness for bacterial physiology and survival. 

Unintentionally, we found that overexpression of *flhDC* genes could inhibit bacteria growth, and strong overexpression of *flhDC* genes from pN15E6 plasmid could lead to *E. coli* host cell death. To elucidate the mechanism behind this phenomenon, we performed transcriptome analysis on *flhDC* over-expressed bacteria to reveal the lethality mechanism.

## 2. Results

### 2.1. Overexpression of flhDC Can Be Lethal to Host Hacteria

**Figure 1:**
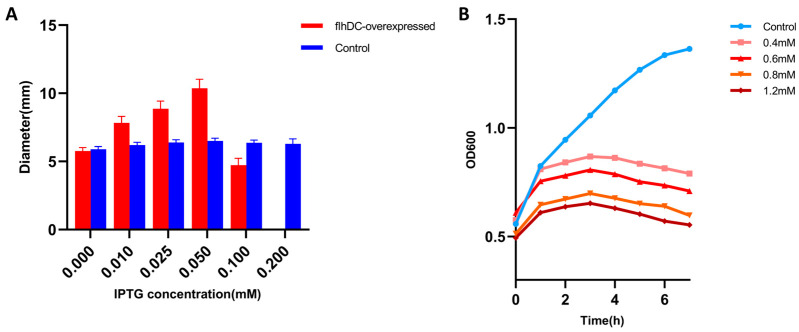
Overexpression of *flhDC* can be lethal to host bacteria. (**A**) The colony diameter of *flhDC-E. coli* recombinant bacteria on the soft agar plate (red bars). *E. coli DH31soplacI* strain as control (blue bars). (**B**) *flhDC* overexpression was induced by the addition of different concentrations of IPTG to the liquid medium of the *flhDC-E. coli* recombinant bacteria and the OD_600_ values were measured at different times after the induction, and it can be found that the growth of the bacteria was inhibited after the addition of IPTG(pink, red, orange, and brown curves) and that the growth curves appeared to decline at 3 h after induction, indicating that the bacteria were dying and lysing.

We cloned the *flhDC* genes into the pN15E6 plasmid [33], which has a robust regulatory ability. We found that the *flhDC*-*E. coli* recombinant bacteria (recombinant *Escherichia coli* strain DH31soplacI transformed with plasmid *flhDC*-*pN15E6*) could grow on the plate with kanamycin (kan) and chloramphenicol (chl) without Isopropyl-beta-D-thiogalactopyranoside (IPTG) induction. However, it could not grow on kan and chl plate containing inducer (0.4 mM IPTG). We induced *flhDC* gene expression with different concentrations of IPTG and determined the diameter of the colony grown on a 0.5% soft agar plate with kan and chl. Figure 1A showed that the diameter of the colonies increased when the concentration of IPTG increased at a low level, indicating that the increased expression of *flhDC* enhanced bacterial motility. However, the diameter of the colonies decreased and eventually, no bacteria grew, after the concentration of IPTG reached a certain concentration, as compared to *E. coli* harboring pN15E6 empty vector alone (control). 

We also induced *flhDC* gene expression by different concentrations of IPTG in a liquid LB medium and determined the OD_600_ value at different time points to judge bacteria growth. We found that the *flhDC*-*E. coli* recombinant bacteria showed growth inhibition as they were induced with IPTG, and the inhibition was more severe as the IPTG concentration increased, as compared to *flhDC*-*E. coli* recombinant bacteria without IPTG induction. Besides, the growth curve shows that OD_600_ started to drop at 3 h after induction (Figure 1B), and flocculent precipitates appeared in the liquid medium as OD_600_ decreased, indicating the debris of dead and lysed bacteria.

To explore the death mechanism, we used transcriptome analysis, referred to data above, the growth and induction parameters were chosen as *flhDC*-*E. coli* recombinant bacteria were grown to an OD_600_ of 0.5 with agitation in LB liquid medium, and then IPTG was added to a concentration of 0.6 mM and induced for a further 2.5 h, meanwhile, *flhDC*-*E. coli* recombinant bacteria without IPTG and incubated for the same time were used as control. Bacterial cells were then harvested and RNAs were prepared for further analysis.

### 2.2. Analysis of Differentially Expressed Genes

We used bacteria that had been induced by 0.6 mM IPTG for 2.5 h (supposed to be just before death) for transcriptome sequencing and *flhDC*-*E. coli* recombinant bacteria incubated for the same time without IPTG induction was used as a control. A total of 31,367,724 raw reads (control group: 1,5013,008, *flhDC* over-expressed group: 16,354,716) were obtained from RNA-seq raw data. After removing low-quality reads and reads with adaptor sequences, 30,750,508 clean reads (control group: 14,770,190, *flhDC* over-expressed group: 15,980,318) were obtained. We then mapped using Bowtie2, for control and *flhDC* over-expressed samples, 14,396,239 and 13,683,571 reads were mapped to the reference genome with mapped rates of 97.47% and 85.63%, respectively. Among the matched 4498 target genes for the control group, 4027 with FPKM ≥ 1; For *flhDC* over-expressed group, 4498 target genes were matched and 4115 with FPKM ≥ 1, as shown in Table 1.

**Figure 2:**
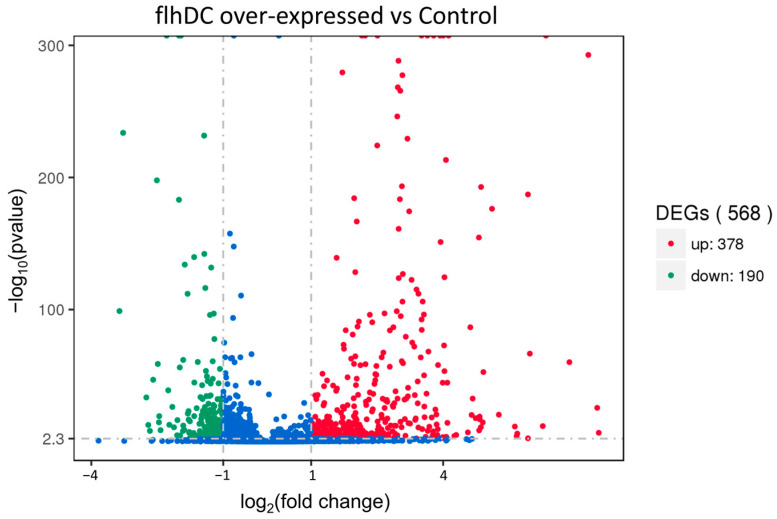
Volcano map of differentially expressed genes between *flhDC* over-expressed group and control group. Red spots represent significantly up-regulated genes, green spots represent significantly down-regulated genes and blue spots represent no significant differentially expressed genes.

A total of 568 DEGs were identified by RNA-Seq, and the volcano plot depicts the statistically significant relationship between the change in expression ploidy and the difference in expression of DEGs, with red and green spots, indicating up- and down-regulated DEGs, respectively (*p* < 0.05), and blue spots indicate non-significant, as shown in Figure 2.

### 2.3. Functional Enrichment Analysis of DEGs

**Figure 3:**
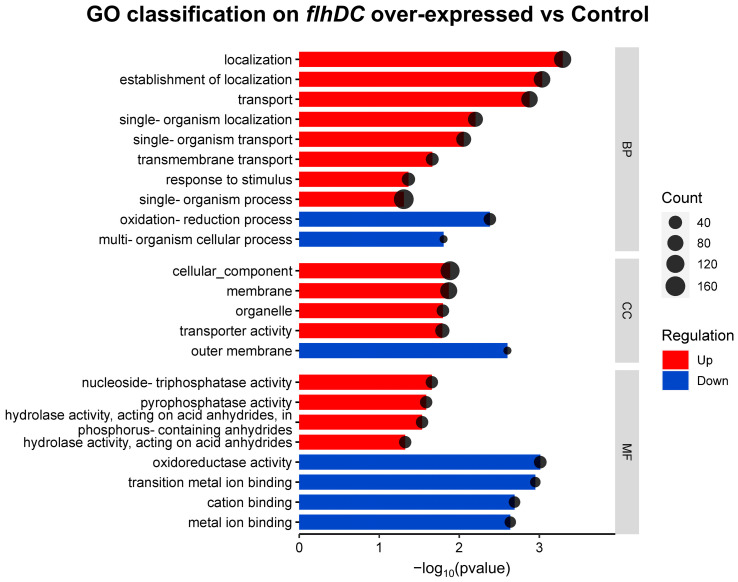
GO functional enrichment of differentially expressed genes between *flhDC* over-expressed group and control group. The size of the spots indicates the number of genes enriched, with red bars indicating up-regulated enrichment and blue bars indicating down-regulated enrichment.

To further understand the functions of the DEGs, we compared and classified the DEGs with the Gene Ontology (GO) database. Figure 3 shows the main categories: biological processes, cellular components, and molecular functions. It could be found that the up-regulated DEGs were mainly concentrated under the classification of biological processes, and the more enriched functions were localization, transport, and locomotion, the more enriched functions in cellular components were cell projection and bacterial-type flagellum, molecular functions were enriched in relatively few genes. The down-regulated DEGs were mainly concentrated in molecular functions, with more enriched functions in oxidoreductase activity, cation binding, and metal ion binding. The more enriched functions in biological processes were the oxidation-reduction process and multi-organism cellular process, and the more enriched functions in cellular components were the outer membrane.

**Figure 4:**
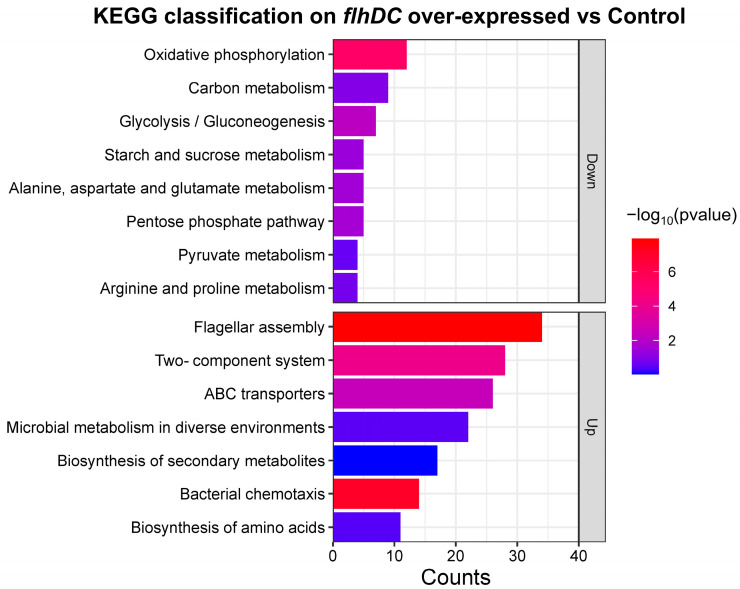
KEGG pathway enrichment of differentially expressed genes between *flhDC* over-expressed group and control group. The horizontal axis indicates the number of genes enriched under the pathway. The colors of the columns indicate significance.

Pathway enrichment analysis was performed based on the Kyoto Encyclopedia of Genes and Genomes (KEGG) database to reveal DEGs’ metabolic and signaling pathways. As shown in Figure 4, among the enriched pathways, significant enrichment was observed in the regulation of flagellar assembly, bacterial chemotaxis, two-component systems, ABC transporter, oxidative phosphorylation, glycolysis/gluconeogenesis and starch and sucrose metabolism. The enrichment results indicated that the overexpression of *flhDC* mainly affected pathways related to energy metabolism, biosynthesis and transmembrane transport.

**Figure 5:**
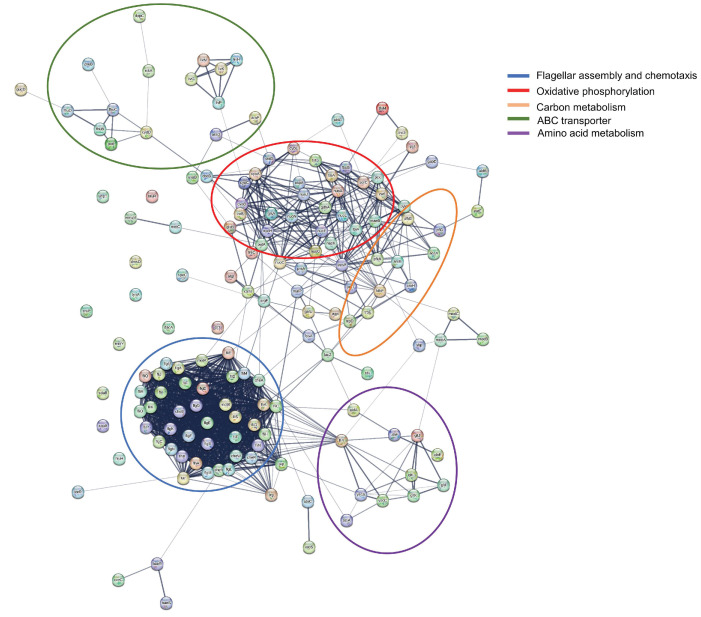
PPI analysis of DEGs in the selected pathways. Different colored circles stand for gene clusters of different functions. The blue, red, yellow, green and purple circles represent flagellar assembly and chemotaxis, oxidative phosphorylation, carbon metabolism, ABC transporter, and amino acid metabolism, respectively.

STRING analysis was used to explore the potential interaction network of the DEGs. The 378 up-regulated DEGs and 190 down-regulated DEGs were analyzed by PPI net, respectively. The DEGs were mainly clustered into flagellar assembly, bacterial chemotaxis, oxidative phosphorylation, carbon metabolism and amino acid metabolism, this was similar to the KEGG enrichment results. Then we analyzed the DEGs of flagellar assembly, oxidative phosphorylation and carbon metabolism, Figure 5 showed that most of them had a relationship with each other, and *fliY*, *nuoC*, *putA*, *pfkA*, and *cbdB* were hub genes. In addition, not all DEGs showed a connection with others because their functions were either unrelated or have not yet been clarified.

### 2.4. Quantitative Real-Time PCR Validation

**Figure 6:**
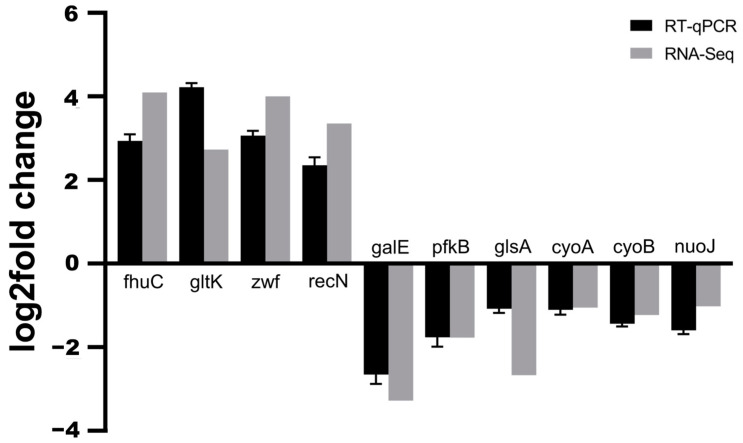
The RT-qPCR verification of differentially expressed genes between the *flhDC* over-expressed group and control group (*flhDC*-*E. coli* recombinant bacteria without IPTG induction). Horizontal axis stands for different genes and vertical axis stands for gene differential expression levels.

To validate the reliability of transcriptome data by RNA-seq, ten DEGs were randomly selected from different pathways (*galE*, *fhuC*, *gltK*, *pfkB*, *glsA*, *zwf*, *recN*, *cyoA*, *cyoB*, and *nuoJ*) for RT-qPCR analysis. In general, the RT-qPCR results were in accordance with the sequencing results, indicating that the transcriptome data were reliable (Figure 6).

### 2.5. Excessive Expression of the Oxidative Phosphorylation Pathway Is the Primary Cause of Bacterial Eell Death

**Figure 7:**
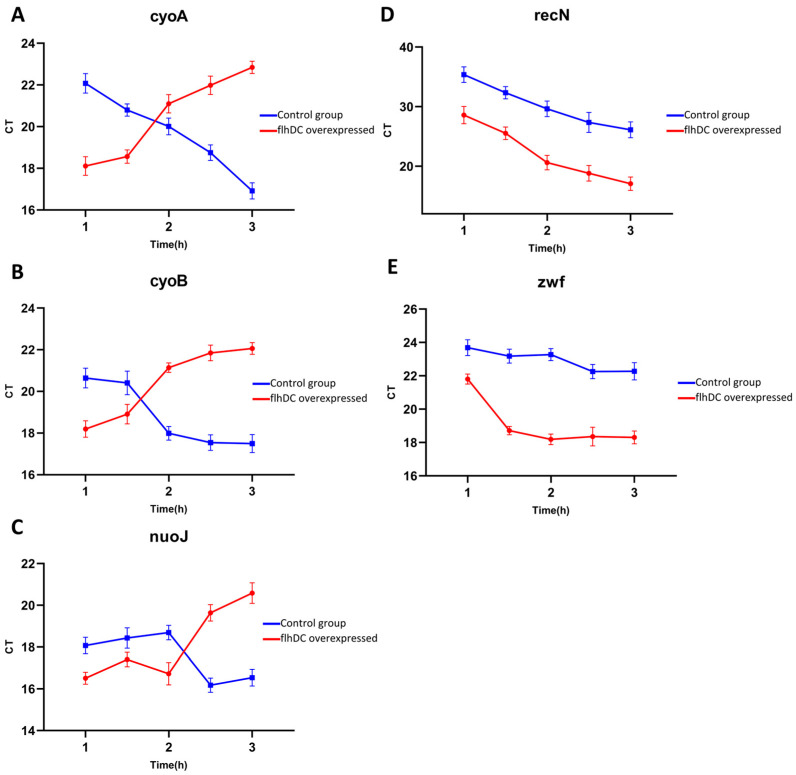
Characterization of temporal expression of different genes using RT-qPCR. RT-qPCR was used to determine the expression levels of several genes after being induced by IPTG, the horizontal axis stands for the time of IPTG induction, and the vertical axis stands for the Ct value, the smaller the Ct value is, the higher the expression levels are. (**A**–**C**) Expression levels of the oxidative phosphorylation genes *cyoA*, *cyoB* and *nuoJ*. (**D**) Expression levels of the DNA damage recognition gene *recN* at different times. (**E**) Expression levels of *zwf* gene at different times. The red curve indicates IPTG-induced *flhDC*-*E.coli* recombinant bacteria, the blue curve indicates *flhDC*-*E. coli* recombinant bacteria without IPTG induction.

Flagellar synthesis and motility are very energy-consuming activities. Therefore, when *flhDC* was highly over-expressed artificially, flagellar synthesis and motility are greatly enhanced, which inevitably leads to an excessive energy requirement, and should eventually lead to elevate the oxidative phosphorylation process enormously. However, the transcriptome results of KEGG enrichment showed that oxidative phosphorylation was down-regulated but not up-regulated instead in RNA-seq data (i.e., at the sampling moment for RNA-seq). We speculated that the down-regulation might be a compromise of strong feedback regulation from the side product at that late stage. To verify this possibility of our speculation, we selected several key enzyme genes of respiratory chain complex I in the oxidative phosphorylation process to quantify *nuoJ*, *cyoA*, and *cyoB*. Their expression was quantified with RT-qPCR in bacteria induced by IPTG and sampled at different time points. Figure 7A–C show that their expression in the *flhDC* over-expressed group was significantly elevated, compared to the control group at the early stage, and then decreased afterward at the late stage. These results indicated that oxidative phosphorylation was indeed enhanced but only in the early stage. The RT-qPCR results also supported the RNA-seq results and showed that the bacteria chose to down-regulate oxidative phosphorylation-related genes at a late stage, perhaps to maintain intracellular homeostasis continually as a compromise.

### 2.6. Excessive Oxidative Phosphorylation Leads to High ROS Production

ROS is a by-product of ATP production [34] and most of the enzymes primarily associated with ROS production are related to respiratory chain complex Ⅰ [35,36]. Thus, the excessive oxidative phosphorylation at an early stage should lead to excessive production of ROS [37].

**Figure 8:**
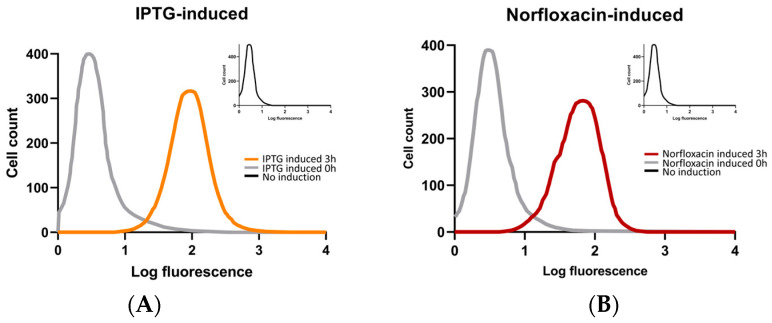
ROS levels and cellular distribution. (**A**) The fluorescence peaks of *flhDC-E. coli* after 3 h induction by IPTG. The gray line is the fluorescence peak at 0 h before induction, the black line (at corner upright) is that at 3 h without induction, and the orange line is that at 3 h of induction by IPTG. (**B**) The fluorescence peaks of *flhDC-E. coli* recombinant bacteria treated with 250 ng/mL norfloxacin c for 3 h. The gray line is at 0 h before induction, the black line (at corner upright) is at 3 h without induction, and the brown line is at 3 h with norfloxacin treatment. The horizontal axis is log_10_ fluorescence intensity, and the vertical axis is cell number.

To verify the over-production of ROS, we stained bacterial cells with DCHF-DA. In addition to *flhDC-E. coli* recombinant bacteria, the norfloxacin-treated bacterial cells were also involved, as positive control, in the experiment. We used flow cytometry to detect cells with fluorescence and graphing showed the level of intracellular ROS and cellular distribution. The flow cytometry results showed that, as compared with negative controls, bacterial cells inducted by IPTG after 3 h had significantly higher fluorescence intensity, which is similar to that treated with norfloxacin for 3 h (Figure 8A,B).

### 2.7. Cellular Response to ROS Damage

Cellular damage occurs when ROS output is beyond the regulation of cellular homeostasis [38]. Transcriptome results showed that the expression of *recN*, which encodes SMC-like proteins (structural maintenance of chromosomes) increased (log_2_fold change = 3.3559). *recN* is the first responder to DNA damage, acting as a sensor that facilitates the orderly recruitment of repair proteins to the injury site and directs the DNA repair response [39]. The up-regulation of *recN* expression indicated that DNA damage had occurred and that the damage repair machinery had recognized the damage and begun mobilizing downstream repair proteins. We also found up-regulation of other genes associated with DNA repair (Table 2) in the RNA-seq result. The expression of *recN* was quantified with RT-qPCR in bacteria induced by IPTG and sampled at different time points, and we found that the expression of *recN* was higher than that of the control group (Figure 7D) and the longer the duration of IPTG induction, the higher the expression of recN.

Besides, we found that the expression of *zwf*, the gene encoding glucose-6-phosphate dehydrogenase (G6PDH), was significantly high in the transcriptome results (log_2_fold change = 4.0027), while the expression of genes related to glucose metabolism was differentially down-regulated (Table 2). The result of RT-qPCR showed that its expression was substantially higher in the *flhDC* overexpression group compared to the control group from the very beginning (Figure 7E). These results above supported the idea that cells were responding to oxidative stress caused by ROS.

## 3. Discussion

In this study, we found that overexpression of the flagellar master regulatory genes *flhDC* was lethal to *Escherichia coli*. Then, we used transcriptome analysis to investigate the mechanism of *flhDC* overexpression using bacteria after 2.5 h of IPTG induction and before cell lysis. By analyzing the functional enrichment results, we targeted the primary cause of lethality of over-expressed *flhDC* to oxidative phosphorylation. We examined the expression of key genes (*cyoA*, *cyoB* and *nuoJ*) of oxidative phosphorylation at different time points by RT-qPCR. Results showed that their expression increased at an early stage and then decreased. These results suggested that the requirement of energy greatly elevated the oxidative phosphorylation processes in the early stage. However, the elevated process had severe side effects and led to overproduction of ROS, which was confirmed using DCHF-DA stain and flow cytometry analysis in this study. The high level of ROS constitutes harm to and should be a strong feedback signal in the subsequent regulation. Our hypothesis above can reasonably explain the lethality phenomena and the transcriptome results obtained.

We noticed that the mechanism of bacterial lethality by *flhDC* overexpression is highly similar to the bactericidal effect of antibiotics. Bactericidal antibiotics share a common mechanism of cell death by forming harmful ROS stimulated by metabolism-related NADH depletion, resulting in iron leaching of iron-sulfur clusters and the Fenton reaction [40]. Michael A et al. observed a significant decrease in the susceptibility of these bacteria to norfloxacin, ampicillin, and kanamycin following the inhibition of ROS generation [41]; furthermore, the effectiveness of antibiotics in killing these bacteria was notably diminished. These findings provide compelling evidence for the essential association between ROS and antibiotic-induced cell death, underscoring the crucial role of ROS in the bactericidal mechanism of antibiotics. Kohanski et al. identified genes commonly up- or down-regulated by the antibiotics norfloxacin, ampicillin, and kanamycin. By comparison, several genes (*nuoC*, *nuoE*, *nuoF*, *xerC*, *nudC*, and *torR*) were the same as found in this research, and notably, most of the genes with oxidative phosphorylation functions in the transcriptome results encoded respiratory chain complex I (*NuoACGFHJLN* and *cyoABCE*), which thought to be a significant target common to antibiotics [42,43]. This similarity suggested that overexpression of *flhDC* had a similar killing mechanism as antibiotics.

ROS can induce DNA damage, posing a significant risk to intracellular homeostasis across all organisms. In the initial stages of damage response, cells undergo modifications to their chromatin structure (or nucleoid in bacteria) and employ specialized repair pathways to eliminate DNA lesions [44]. In cases where the damage remains unaddressed, a complex cascade of interconnected signaling reactions, referred to as the DNA damage response (DDR), orchestrates cell cycle arrest, activation of transcriptional programs, and initiation of DNA repair processes [45]. We verified that the ROS output levels in the *flhDC* over-expressed and norfloxacin-treated group were indeed higher than those in the control group by flow cytometry. Under normal conditions, an appropriate amount of intracellular ROS maintains and mediates normal intracellular signaling pathways and normal physiological functions [46,47], but excessive production or abnormal sources of ROS can lead to oxidative stress, which can lead to genetic mutations, induce irreversible oxidative modifications of proteins, lipids, and polysaccharides, impair their function, cause pathological damage and promote disease development or cell death [48]. *recN* is thought to be the first responder to DNA damage, acting as a sensor that facilitates the orderly recruitment of repair proteins to the site of injury and directs the repair response [49]. Pierre et al. found that treatment of Bacillus subtilis cells with peroxide resulted in a significant increase in *recN* expression [50], which is similar to what we found in *flhDC* over-expressed *E. coli*.

The pentose phosphate pathway is essential for maintaining redox homeostasis under oxidative stress, and NADPH is required as a redox equivalent in the antioxidant mechanism [51]. Glucose 6-phosphate dehydrogenase (G6PDH) catalyzes the conversion of glucose 6-phosphate to gluconolactone 6-phosphate. This process produces NADPH and is the primary output pathway of NADPH. Thus, the G6PDH is the key enzyme in the antioxidant reaction. The expression of *zwf* (the gene encoding G6PDH) is regulated by the NADPH/NADP^+^ ratio [51,52]. We verified that the *zwf* gene appeared significantly upregulated in *flhDC* overexpression cells (Figure 7E). In addition, we found that some other genes related to glucose metabolism were differentially down-regulated (Table 2). This is because PPP activity is rapidly increased in reaction to oxidative stress, and the cell’s metabolism is adaptive to guarantee that the metabolic network functions stabilize in response to the stress [53,54]. This tight regulation appears to have a dual role. It inhibits the overproduction of NADPH and PPP intermediates during normal growth and reduces carbon depletion owing to CO_2_ production. At the same time, it promotes a quick cellular response while under stress [55,56]. To simultaneously preserve resources and energy, maintain homeostasis in the cell, and avoid the collapse of the metabolic network, the adaptations include raising the quantities of necessary components and decreasing the concentration of undesirable components.

## 4. Materials and Methods

### 4.1. Strains and Plasmids

The *Escherichia coli DH31soplacI* strain and pN15E6 plasmid were kindly provided by Professor Ravin NV [33]. This plasmid has a double *lac* operator suppression module on the phage *T5* promoter, allowing separate control of promoter activity and copy number. The *flhDC*-pN15E6 plasmid is a derivative of pN15E6 with *flhDC* genes cloned downstream of promoter *PT5-lac* and was constructed in our laboratory. The *flhDC-E. coli* recombinant bacteria are *Escherichia coli DH31soplacI* transformed with the *flhDC*-pN15E6 plasmid.

### 4.2. Growth of flhDC-E. coli Recombinant Bacteria on Soft Agar and LB Liquid

A colony of *flhDC*-*E. coli* recombinant bacteria and of *E. coli* harboring pN15E6 empty vector alone were inoculated into fresh LB liquid with kanamycin (15 µg/mL) and chloramphenicol (11 µg/mL) and the culture was incubated at 37 °C with agitation overnight. To determine bacterial motility, a drop of 1 µL of *flhDC*-*E. coli* and of E. coli harboring pN15E6 empty vector alone overnight cultures was carefully dipped on a soft agar plate and the plate was incubated for a further 6 h. The colony diameter was measured afterward. The results were then plotted in Figure 1A.

Fresh LB liquid with kan and chl was inoculated with *flhDC*-*E. coli* recombinant bacteria overnight cultures at 2%. The culture grew at 37 °C with agitation until the OD_600_ reached 0.5. IPTG (Shanghai Shenggong Bioengineering Co., Shanghai, China.) was added to a final concentration of 0.6mM for the induction of *flhDC* overexpression. The culture grew continually at 37 °C with agitation and the OD_600_ was measured every hour until seven hours after induction. The growth curve was then plotted in Figure 1B.

### 4.3. RNA Extraction, Library Preparation, and Sequencing

Total RNA was extracted from cells induced with IPTG or without induction (control bacteria) using FastPure^®^ Cell/Tissue Total RNA Isolation Kit (Vazyme, Nanjing, China) according to the manufacturer’s instructions.

The cDNA library construction and sequencing were performed at the Beijing Novogene Biotech Co. Ltd. (Beijing, China). Using a fragmentation buffer, the mRNA was cut up into tiny bits. Using an mRNA template and the SuperScript II Reverse Transcriptase kit, the first-strand cDNA was created. (Thermo Fisher Scientific, Waltham, MA, USA). The SuperScript Double-Stranded cDNA Synthesis kit was then used to create the second-strand cDNA. (Thermo Fisher Scientific, Waltham, MA, USA). After end repair, poly (A) addition, and adapter ligation, agarose gel electrophoresis was used to identify fragments of the right size for PCR amplification. Using the Illumina HiSeq technology, the final cDNA library was sequenced, yielding 150 bp paired-end reads.

### 4.4. Bioinformatic Data Analysis

Low-quality reads, reads including adapters, and ambiguous “N” bases higher than 5% were then eliminated from the raw readings data to produce clean reads. The superior clean readings were mapped using the Bowtie2 program. Then, the gene expression level was evaluated using RSEM v1.3.3 software, and it was further standardized using the FPKM technique. The DESeq2 was used for the differential expression analysis, and the false discovery rate (FDR) was used as the *p*-value cutoff. After comparing the FPKM data, fold changes between the treatment and control groups were computed. The threshold for differentially expressed genes (DEGs) was set at *p* < 0.05 with fold changes 2 as the upper limit.

### 4.5. Differential Expression Genes (DEGs) Analysis and Annotation

To identify DEGs between the two samples, the expression level for each transcript was calculated using the FPKM (the fragments per kilobase of exon per million mapped reads) method. Cuffdiff (http://cufflinks.cbcb.umd.edu/, URLs accessed on 20 February 2018) was used for differential expression analysis and the DEGs were selected using the following criteria: the logarithmic of fold change was greater than 2 and the *p*-value should be less than 0.05.

### 4.6. Functional and Protein-Protein Interaction (PPI) Analysis of DEGs

To understand the functions of the DEGs, Gene Ontology (GO) functional enrichment and Kyoto Encyclopedia of Genes and Genomes (KEGG) pathways enrichment were carried out by ClusterProfiler (3.8.1). DEGs were significantly enriched in GO terms and KEGG pathways when their *p*-value was less than 0.05.

### 4.7. Reverse Transcription and Quantitative Real-Time RT-PCR (qRT-PCR)

The extracted RNAs were reverse-transcripted to complementary DNA (cDNA) by a reverse transcriptase synthesis kit (Vazyme, Nanjing, China). The primers were designed using Oligo7 and primer information was detailed in Table 3. The RT-qPCR amplification was carried out with ChamQ Universal SYBR qPCR Master Mix (Vazyme, Nanjing, China). The program was set as 95 °C for 30 s, 95 °C for 15 s, 62 °C for 30 s, 72 °C for 15 s, for 40 cycles. The results were analyzed using the 2-ΔΔCT method with U16SRT as a reference gene.

### 4.8. ROS Detection by Flow Cytometry

An amount of 50 mL LB was inoculated with 2% overnight of *flhDC-E. coli* recombinant bacteria. The culture was incubated at 37 °C with vigorous agitation until OD_600_ reached about 0.5. IPTG was added to a final concentration of 0.6 mM to induce overexpression of *flhDC* from the *flhDC*-pN15E6 plasmid. As a positive control, norfloxacin was added to a parallel culture to a final concentration of 250 ng/mL, to kill the bacteria. The cultures were incubated for another 3 h. Bacterial cells were harvested by centrifugation. Then DCFH-DA (with a final concentration of 10 μmol/L) was added to the harvested bacterial cells suspended in 600 μL buffer and incubated at 37 °C for a further 15 min. The cells were then collected by centrifugation and washed three times with PBS buffer to remove free dye. Samples were subsequently assayed using flow cytometry, the excitation wavelength was 488 nm and the emission wavelength was 525 nm.

## Figures and Tables

**Table 1:** Summary of reads quality and mapping results of RNA-Seq.

	Sample	*flhDC* Over-Expressed	Control
Parameter			
Total raw reads		16,354,716	15,013,008
Total clean reads		15,980,318	14,770,190
Mapped reads		13,683,571	14,396,239
Mapped rate (%)		85.63%	97.47%
FPKM 0−1		383 (8.51%)	471 (10.47%)
FPKM 1−3		221 (4.91%)	324 (7.20%)
FPKM 3−15		777 (17.27%)	773 (17.19%)
FPKM 15−60		1135 (25.23%)	1018 (22.63%)
FPKM > 60		1982 (44.06%)	1912 (42.51%)

**Table 2:** Genes related to DNA repair and glucose metabolism.

Genes’ Name	log_2_fold Change	Description
*recN*	3.3559	DNA repair protein
*recJ*	2.0536	single-stranded-DNA-specific exonuclease
*held*	1.1701	DNA helicase IV
*torR*	2.1721	DNA-binding response regulator
*sbmC*	4.8629	DNA gyrase inhibitor
*rsgA*	1.7846	Small ribosomal subunit biogenesis GTPase
*zwf*	4.0027	Glucose-6-phosphate dehydrogenase
*pfkB*	−1.7747	6-phosphofructokinase
*pykF*	−1.0239	pyruvate kinase
*ppsA*	1.1415	phosphoenolpyruvate synthase
*pck*	−1.0299	phosphoenolpyruvate carboxykinase
*fbaB*	−1.2847	fructose-diphosphate aldolase
*maeB*	1.7846	malic enzyme

**Table 3:** Amplification primers of target genes.

Genes’ Name	Sequence (5′-3′)		Length
*cyoA*	Forward	GTTCACTGATACTGACGGCAT	121 bp
	Reverse	TTCGGGCTGTACTTAGCATC	
*cyoB*	Forward	TGGCCTGATCACTTACTTCGG	138 bp
	Reverse	ATAATGGCGTCAGCAAAACCAC	
*nuoJ*	Forward	GCCATCCTCGGTGTTAACGAT	114 bp
	Reverse	CAGCATAGAAGCCAGTTCCAC	
*fhuC*	Forward	TCTTGATGCCCAACCGCTGGA	138 bp
	Reverse	CCATGCCACGGGTAACGACCA	
*gltK*	Forward	GGTTTGCCAAAGCCTACGTT	111 bp
	Reverse	GCGATAATCCCAGCACGTT	
*glsA*	Forward	GTGGATCAGGCTTACACCCAA	140 bp
	Reverse	TCACCCGCACTATAGACGTTG	
*zwf*	Forward	CCAGGTTTACCGTATCGACCAC	112 bp
	Reverse	ATCAATGGTGCGATTGTCCC	
*galE*	Forward	AAGGCATTCCGAATAACCTGA	100 bp
	Reverse	CCATCTTCGGTCGGATAATCGTT	
*pfkB*	Forward	ATGAAAATGTCCCCGTCGCTAC	198 bp
	Reverse	GCAGGCTTCCGCTTATGACCA	
*recN*	Forward	GCCTATTGCCAAAGTCGCATC	124 bp
	Reverse	CTGTTGGACCGCTAATCCCT	

## Data Availability

All data generated during this study are included in this article. RNASeq data have been uploaded to https://github.com/SimonSun131/RNA-Seq-DATA.git (accessed on 13 August 2023).
